# Stiffness and ultimate load of osseointegrated prosthesis fixations in the upper and lower extremity

**DOI:** 10.1186/1475-925X-12-70

**Published:** 2013-07-11

**Authors:** Bastian Welke, Christof Hurschler, Marie Föller, Michael Schwarze, Tilman Calliess

**Affiliations:** 1 Laboratory for Biomechanics and Biomaterials, Department of Orthopaedics, Hannover Medical School, Anna-von-Borries-Str. 1-7, 30625 Hannover, Germany; 2 Department of Orthopaedics, Hannover Medical School, Anna-von-Borries-Str. 1-7, 30625 Hannover, Germany

**Keywords:** Transfemoral amputation, Transhumeral amputation, Osseointegration, Skeletal attachment, Stiffness, Ultimate load, In vitro, Bending, Axial pull-out, Human bones, Synthetic bones

## Abstract

**Background:**

Techniques for the skeletal attachment of amputation-prostheses have been developed over recent decades. This type of attachment has only been performed on a small number of patients. It poses various potential advantages compared to conventional treatment with a socket, but is also associated with an increased risk of bone or implant-bone interface fracture in the case of a fall. We therefore investigated the bending stiffness and ultimate bending moment of such devices implanted in human and synthetic bones.

**Methods:**

Eight human specimens and 16 synthetic models of the proximal femora were implanted with lower extremity prostheses and eight human specimens and six synthetic humeri were implanted with upper extremity prostheses. They were dissected according to typical amputation levels and underwent loading in a material testing machine in a four-point bending setup. Bending stiffness, ultimate bending moment and fracture modes were determined in a load to failure experiment. Additionally, axial pull-out was performed on eight synthetic specimens of the lower extremity.

**Results:**

Maximum bending moment of the synthetic femora was 160.6±27.5 Nm, the flexural rigidity of the synthetic femora was 189.0±22.6 Nm^2^. Maximum bending moment of the human femora was 100.4±38.5 Nm, and the flexural rigidity was 137.8±29.4 Nm^2^. The maximum bending moment of the six synthetic humeri was 104.9±19.0 Nm, and the flexural rigidity was 63.7±3.6 Nm^2^. For the human humeri the maximum bending moment was 36.7±11.0 Nm, and the flexural rigidity at was 43.7±10.5 Nm^2^. The maximum pull-out force for the eight synthetic femora was 3571±919 N.

**Conclusion:**

Significant differences were found between human and synthetic specimens of the lower and upper extremity regarding maximum bending moment, bending displacement and flexural rigidity. The results of this study are relevant with respect to previous finding regarding the load at the interfaces of osseointegrated prosthesis fixation devices and are crucial for the development of safety devices intended to protect the bone-implant interface from damaging loadings.

## Background

The conventional treatment for individuals with an amputation is the attachment of prosthesis by means of a socket over the amputated limb. As an alternative, direct skeletal attachment of prostheses has been developed over recent decades. This type of attachment has only been performed on a small number of patients (e.g., the Osseointegrated Prosthesis for the Rehabilitation of Amputees (OPRA) device has been reported for over 100 patients) [1,2]. This technique involves the connection of a prosthesis directly to the skeletal system, circumventing the known issues of conventional socket attachment, such as skin and soft tissue irritation and a lack of osseoperception [3-6]. The outcome indicates an improvement in the individual’s quality of life, mostly due to increased prosthetic use without an increase in prosthetic related problems [7]. However, the indication for the use of this technique is still limited due to unresolved complications. Although it has been possible to reduce complications by optimization of treatment protocols, strict patient selection and modifications of the implant design, revision rates of about 50% are still reported [6,8]. At present, research in this area is focused primarily on the minimization of existing disadvantages, such as latent infection at the skin-implant interface and the risk of periprosthetic fractures triggered by a fall of the patient. The consequences of such a periprosthetic fracture often leads to revision surgery or a complete removal of the skeletal attachment system and return to conventional treatment. Such a scenario is associated with a high level of morbidity related to lengthy hospitalization and loss of mobility following the reapplication of a conventional socket treatment.

The increased risk of falling of individuals with trans-femural amputation has been demonstrated and poses a risk for both the upper and lower extremity. Typical falling scenarios [9] generate bending moment as predominate loads [10-12] which could lead to periprosthetic fractures [13] and implant failure [14]. Risk analysis and self-reported questionnaires concerning additional loads have identified axial pulling forces as likely to occur while catching one’s foot [15]. Only a few studies have dealt with the impact and injury risk to the upper extremity [16]. Fall arrest strategies that involve bracing the body with the hands may generate considerable bending moment loads [17].

To author’s knowledge, the only biomechanical test concerning stability of an osseointegrated prosthesis fixation reported in the literature was limited to torsional loads of the lower extremity [18]. To prevent fractures from these loads, safety devices (e.g. Rotasafe [19] or internal predetermined breaking points [18]) have been developed and are in use by some patients. To date, the sensitivity of osseointegrated prosthesis fixation to bending moments and axial pull-out forces has not been investigated and is not addressed by current safety devices.

The objective of this *in vitro* study was thus to determine the mechanical properties and failure modes of an osseointegrated prosthesis fixation for both the lower and upper extremities in human and synthetic models.

## Methods

Synthetic bones are often used as substitutes for human specimens in biomechanical studies, because they display good agreement with macroscopic mechanical and geometrical properties [20] of physiological bone, because they are standardized and because they are regarded as cost effective in comparison to cadaver tissue.

### Specimens - lower extremity

Eight human specimens and 16 synthetic models of the proximal femora were used. The median age of the human donors was 73.5 years (Institute for Functional and Applied Anatomy, Hannover Medical School, Hannover, Germany). Ethical approval was given by the local committee (No. 1864–2013). Bone density was measured in the diaphysis using Dual-Energy X-ray Absorptiometry (DEXA) measurement (DEXA HOLOGIC Discovery A), and mean values of 1.59±0.17 g/cm^2^ were observed, which is above values for a comparable group without bone-related diseases [21]. The human specimens were fresh-frozen at -22°C prior to testing. The synthetic femora models were medium-sized and had left-side geometry (#3403, 4^th^ generation, Sawbone, Pacific Research Laboratories Inc., Vashon, WA, USA).

### Specimens – upper extremity

Eight human specimens and six synthetic humeri were used. The human specimens were taken from the same donors as the femora specimens, bone density measured in the diaphysis by DEXA was observed to be 0.94±0.2 g/cm^2^, which was slightly lower than in a group of healthy young males [22]. The synthetic humeri models were large-sized and had left-side geometry (#3404, 4^th^ generation, Sawbone, Pacific Research Laboratories Inc., Vashon, WA, USA).

### Amputation – lower extremity

An osteotomy was performed on the synthetic femora 220 mm distal from the apex of the greater trochanter. For later embedding, the femoral head was removed. Resection of the human bones was based on the individual anatomy of each specimen. This was determined on plain radiographs by selecting the size and position of the implant in order to achieve complete cortical alignment. Preparation of the samples for implantation was performed according to the manufacturer's standard protocol. After amputation, specimens were treated with a femoral intramedullary stem in cementless design (MUTARS® Implantcast, Germany). Different implant sizes (12, 14, 15, 17 or 18 mm) were used, depending on the individual bone anatomy, to achieve a press-fit implant fixation. In the synthetic femora, the cemented versions of the tumor system were implanted (MUTARS®, size 11, Implantcast, Germany). The major difference between the utilized implant and established osseointegrated fixation devices such as ISP (Eska Implants, Germany) is the design of the transcutaneous region while skeletal anchorage is similar.

The proximal end of each prepared bone specimen was embedded centrally into a metal shell using cold-curing three component resin (Rencast FC52/53 Isocyanate, FC53 Polyol, Füller DT 082, gössl&pfaff GmbH, Karlskron/Braulach, Germany), the distal part with the implant was directly screwed to an embedded adapter in a metal shell. After implantation, samples were checked for obvious defects prior to testing. The length (*l*) between the two embeddings was measured for the synthetic specimens with 184.1 ± 1.7 mm and for the human specimens with 173.1 ± 21.4 mm. One of the eight human specimens had to be excluded from the group of specimens due to a premature failure of the embedding during four-point bending. Therefore the results from seven femora will be presented in the following.

### Amputation – upper extremity

The synthetic humeri were osteotomised at a defined resection level of 210 mm, measured from the greater tubercle. The resection of the human bones was also based on the individual anatomy of each sample and determined using plain radiographs in order to achieve complete cortical alignment. The embedding protocol was identical to that of the femoral specimens.

The humeral intramedullary stems were all implanted cementlessly (all synthetic: size 11, human: size 10–13 mm, MUTARS® Implantcast, Germany). The length (*l*) between the two embeddings was measured for the synthetic specimens with 197.8 ± 4.0 mm and for the human specimens with 187.8 ± 34.1 mm.

### Mechanical testing

Biomechanical testing was performed on a servohydraulic material testing system (MTS MiniBionix I, Model 858, Eden Prairie, Minneapolis). Four-point bending and axial pull-out test modalities were chosen for the lower extremity specimens, and only four-point bending was performed for the upper extremity specimens.

### Four-point bending test protocol

**Figure 1:**
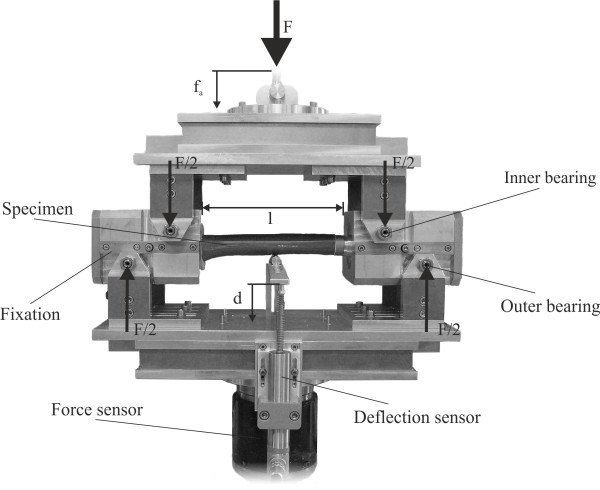
Custom made four-point bending device on the material testing machine (MTS MiniBionix I, Model 858, Eden Prairie, Minneapolis) with a mounted osteotomised synthetic femur with an intramedullary stem.

The specimens were mounted in a custom four-point bending device on the material testing machine (Figure 1), that applies a bending moment in anterior-posterior direction and is constant over the test length of the specimen. The device consisted of a lower part with two fixed outer bearings and an upper part with two inner bearings. The bearings were adjustable to adapt to the different specimen lengths. The displacement of the mid-diaphysis was measured by a lever system and a linear variable displacement transducer (SM407.50.2.X19, Schreiber Messtechnik GmbH, Oberhaching, Germany).

The testing conditions provided no preload and were displacement-controlled at a constant velocity of the upper part of 0.1 mm/s. The termination criterion of the experiment was defined as complete structural failure of the specimen. At every 0.001 mm displacement of the hydraulic cylinder, the following parameters were recorded: time (*t*), axial force (*F*), axial displacement of the upper device (*f*_*a*_) and deflection in the mid-diaphysis (*d*). Based on this data, the flexural rigidity was calculated based on the assumption of a cantilever beam loaded with a constant bending moment M_b_:

$$\mathrm{EI}=\frac{M_{b}l^{2}}{8f_{b}}.$$

True bending was corrected for the influence of the displacement of the upper component of the four-point bending device:

$$f_{b}=d-f_{a}.$$

Flexural rigidity was normalized with respect to length, for improved comparability within human specimens and between the human and synthetic specimens:

$${\left(\mathrm{EL}\right)}_{n}=\frac{M_{b}}{8f_{b}}.$$

The slope of the moment-bending-curve was determined in the linear range, between 25% and 75% of the maximum bending. According to the definition of offset yield strength, the calculated straight line was shifted by a constant offset of 0.2% maximum bending. The intersection between this straight line and the moment-bending curve identified the yield point, which was then used to calculate flexural rigidity.

### Axial pull-out test protocol

**Figure 2:**
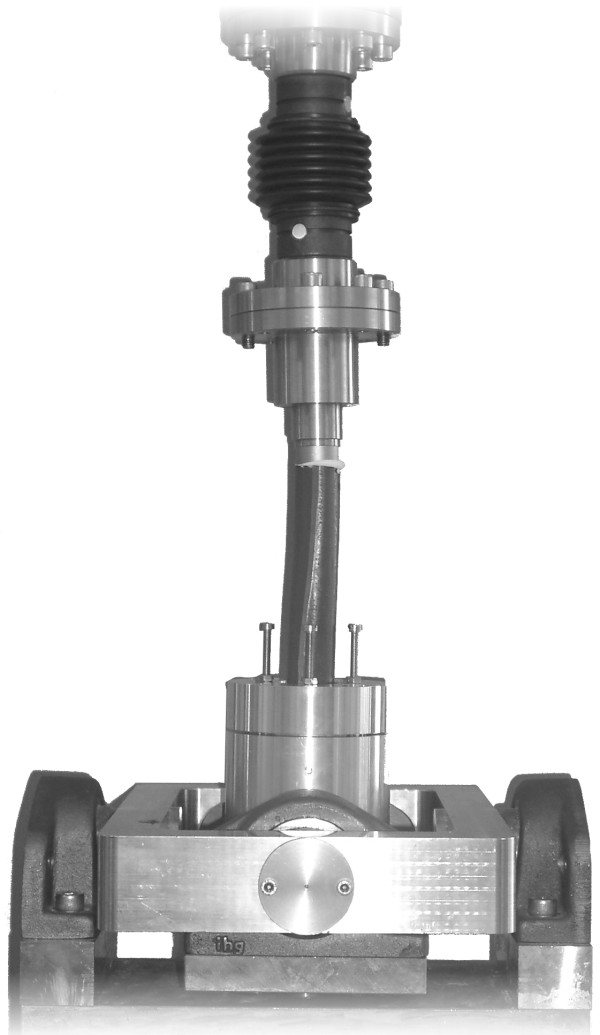
Custom made axial pull-out device on the material testing machine (MTS MiniBionix I, Model 858, Eden Prairie, Minneapolis) with a mounted osteotomised synthetic femur with a cemented intramedullary stem.

The synthetic femora were mounted between two universal-joints to enable a purely tensile load. The distal part of the femur with the intramedullary stem was fixed to the upper machine segment and the embedded proximal part was attached to the lower universal-joint (Figure 2).

A force-controlled preload of F = 0 N applied for a period of 20 s, to minimize the biasing force resulting from mounting the rigid setup. Subsequently, the pull-out test was displacement-controlled with a velocity of 0.1 mm/s. The termination criterion of the experiment was defined as failure of the specimen, which resulted in a decrease in the measured force.

### Statistics

The statistical analysis of the test data was performed with a Mann–Whitney-U-Test as normal distribution of values could not be assumed, and p-values < 0.05 were regarded as statistically significant.

## Results

In total, 22 synthetic and 16 human bones were tested in different loading scenarios. We will first present the results of the biomechanical tests of the lower extremity, followed by the results of the upper extremity.

### Lower extremity - four-point bending

**Figure 3:**
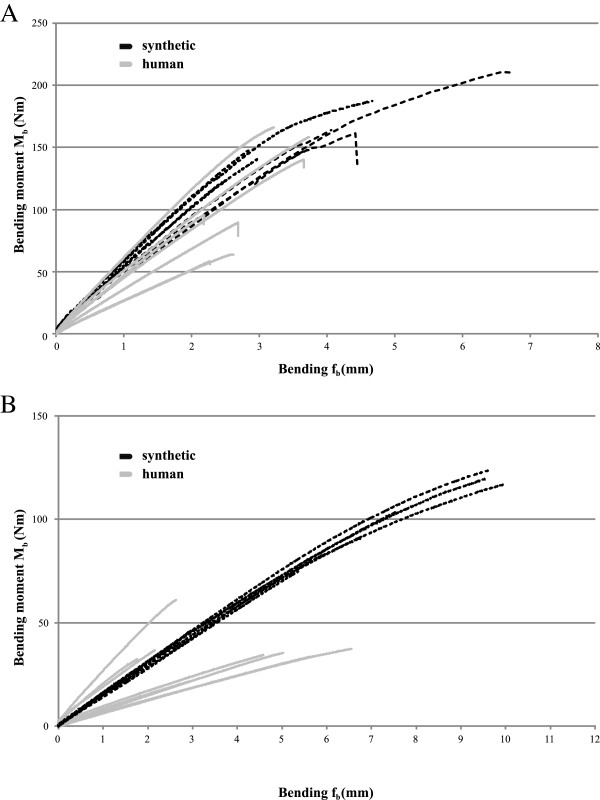
**Relationship between bending moment and true bending from the four-point bending test of the lower (A) and upper (B) extremities.** Comparison of bending moments of synthetic (lower extremity n=8, upper extremity n=6) and human (lower extremity n=7, upper extremity n=8) bones.

The maximum bending moment of the synthetic femora was 160.6±27.5 Nm, and the maximum bending displacement was 4.0±1.3 mm (Table 1 and Figure 3). The flexural rigidity at the offset yield point of the synthetic femora was 189.0±22.6 Nm^2^. Five of the seven synthetic bones were fractured between the minor trochanter and the femoral neck. The other two showed periprosthetic fractures with plastic deformation of the implant stems.

**Table 1:** Mechanical properties of the human (n=7) and synthetic (n=8) lower extremity models as determined by the four-point bending test, and properties of the synthetic (n=8) femora as determined by the axial pull-out test

**Loading scenario**	**Parameter**	**Specimen type**				
		**Human**	**CV**	**Synthetic**	**CV**	**p-value**
**Four-point bending**	Max. moment (Nm)	100.4 ± 38.5	0.38	160.6 ± 27.5	0.17	*0.006*
	Bending at max. moment (mm)	2.7 ± 0.8	0.29	4.0 ± 1.3	0.33	*0.021*
	Flexural rigidity AP (Nm^2^)	137.8 ± 29.4	0.21	189.0 ± 22.6	0.12	*0.009*
	Normalized Flexural rigidity AP (N)	4722.6 ± 1418.4	0.30	5576.2 ± 655.9	0.12	0.232
**Axial pull-out**	Max. axial force (N)	-		3571.4 ± 919.1	0.25	-
	Displacement at max. force (mm)	-		0.5 ± 0.1	0.24	-

Statistical significant differences between human and synthetic bones (alpha=0.05) are marked by italic typeset.

**Figure 4:**
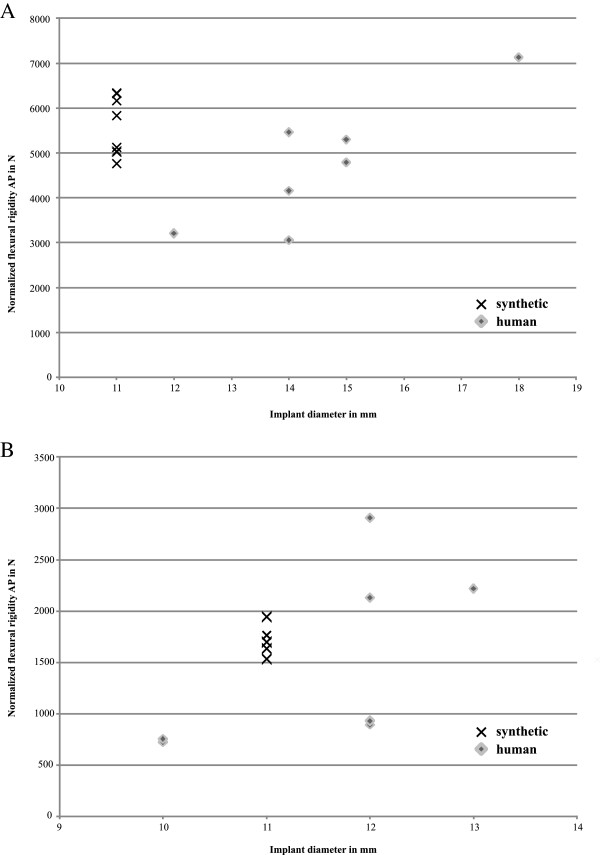
Relationship between normalized flexural rigidity of lower (A) and upper (B) extremity and implant diameter.

The maximum bending moment of the human femora was 100.4±38.5 Nm, and the maximum bending displacement was 2.7±0.8 mm (Table 1 and Figure 3). The shortest bones withstood the highest bending moments. The flexural rigidity at the offset yield point of the human specimens was 137.8±29.4 Nm^2^. In the human specimens, normalized flexural rigidity increased with increasing stem size (Figure 4). Six of the eight bones fractured around the minor trochanter, while the other two showed a longitudinal split along the femoral diaphysis. No stems were damaged during the tests with human specimens.

When comparing synthetic and human femora, significant differences were observed in the following parameters: maximum moment, bending displacement, and flexural rigidity (Table 1). Following normalization of flexural rigidity with respect to specimen length, differences were no longer significant (Table 1). The coefficient of variance (CV) was smaller for the synthetic femora, except for the bending displacement parameter.

### Lower extremity – axial pull-out test

**Figure 5:**
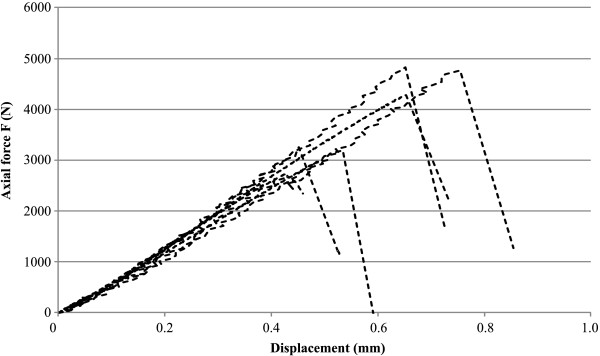
Results from the axial pull-out test of the synthetic (n=8) femora.

The maximum pull-out force for the eight synthetic femora with cemented prostheses was 3571±919 N (Table 1 and Figure 5). In all tested specimens, macroscopically observed relative movement occurred between the implant and the cement. Neither the implants nor the synthetic femora failed.

### Upper extremity – four-point bending

The maximum bending moment of the six synthetic humeri amputation constructs was 104.9±19.0 Nm, and the maximum bending displacement was 8.1±1.9 mm (Table 2 and Figure 3). The flexural rigidity of the synthetic humeri was 63.7±3.6 Nm^2^. Again, two different failure modes could be observed: in three of the specimens, fracture occurred under the minor tubercle, while in the other samples, fracture was within the humeral diaphysis. No implant failure or plastic deformations were observed.

**Table 2:** Mechanical properties of the human (n=8) and synthetic (n=6) upper extremity models as determined by the four-point bending test

		**Specimen type**				
**Loading scenario**	**Parameter**	**Human**	**CV**	**Synthetic**	**CV**	**p-value**
**Four-point bending**	Max. moment (Nm)	36.7 ± 11.0	0.30	104.9 ± 19.0	0.18	*0.001*
	Bending at max. moment (mm)	3.9 ± 1.8	0.45	8.1 ± 1.9	0.23	*0.003*
	Flexural rigidity AP (Nm^2^)	43.7 ± 10.5	0.24	63.7 ± 3.6	0.06	*0.001*
	Normalized Flexural rigidity AP (N)	1434.4 ± 847.5	0.59	1712.8 ± 137.4	0.08	0.491

Statistical significant differences (alpha=0.05) between human and synthetic bones are marked by italic typeset.

For the human humerus specimens, the maximum bending moment was 36.7±11.0 Nm, and the maximum bending displacement was 3.9±1.8 mm (Table 2 and Figure 3). The flexural rigidity at the offset yield point of the human specimens was 43.7±10.5 Nm^2^. In the human specimens, normalized flexural rigidity displayed a tendency to increase with increasing stem size (Figure 4). Three fractures in the region of the minor tubercle, three specimens fail with longitudinal splittings and two diaphysal fractures directly above the implant apex were observed. Again, no failures related to the implants were detected.

In the comparison between the synthetic and human humeri, significant differences were seen for the following parameters: maximal moment, bending displacement and flexural rigidity (Table 2). Analogous to the results of the lower extremity, after normalizing the flexural rigidity, differences were no longer significant (Table 2). In general, the CV for all parameters of the synthetic humeri was lower than the CV of human specimens.

## Discussion

The aim of this study was to determine the mechanical properties of synthetic and human humeri and femora with an established intramedullary stem *in-situ*. Significant differences were found between human and synthetic specimens of the lower and upper extremity regarding maximum bending moment, bending displacement and flexural rigidity. Fracture modes were equally distributed among human and synthetic specimens and showed general agreement.

Contrary to previously reported results utilizing classical four-point bending [20,23], we observed higher maximum bending moments and flexural rigidities for the synthetic specimens of the lower extremity. Gardner et al. found flexural rigidity in anterior-posterior direction to be 32%, while Heiner found it to be 6% higher in human specimens. The previous studies measured structural properties of untreated specimens, while our test setup included an intramedullary stem. The normalized flexural rigidity, where the highly varied length is accounted for, did not show significant differences. The prevailing parameter for structural stiffness, the diameter of the hexagonal stem, showed a proportional influence on normalized flexural rigidity in both the upper and lower extremity human specimens. Normalized flexural rigidity is influenced by the elastic (Young’s) modulus of the material and the area moment of inertia. In this area moment of inertia, the diameter parameter is raised to the fourth power. The increased CV after normalizing flexural rigidity, particularly in the human specimens with varying stem sizes, could be explained by this high power compared to the normalized length parameter, which is raised to the second power in the expression for flexural rigidity. However, the normalized flexural rigidity of the synthetic lower extremity specimens showed more than twofold the values of what might be expected due to stem diameter, indicating a difference in material properties. Interestingly, this was not the case for the upper extremity specimens. Based on the low coefficient of variation for the tested parameters of the synthetic bones it seems reasonable to reduce the number of specimens per group for future *in vitro* studies.

In general, we observed similar fracture modes for the lower and upper extremity for both human and synthetic specimens. One reason for this behavior might be the influence of our specific boundary conditions leading to high stiffness gradients resulting in stress concentrations at the embedding. Despite similar fracture modes, considering the synthetic bones as an appropriate model for an osseointregration fixation in human bone is limited by the highly different bending moments at failure. Interestingly, the observed predominant pertrochanteric fractures in our test setup are consistent with the reported occurrence of real periprostetic fractures in treated population [14].

There are several general limitations of this *in vitro* study that must be acknowledged. The major limitation is the difference in implantation technique between the two groups. Due to the different curvatures of the intramedullary stem and the synthetic bone specimens, as well as the unyielding material properties, it was not possible to establish an appropriate press-fit anchorage and we were forced to revert to a cemented implantation in the synthetic femurs. However, we expect that the 1 mm cement mantle, which leads to an increased effective diameter of the intramedullary stem, would increase the flexural rigidity of the specimen. This was supported by our findings from the lower extremity; when assuming the diameter of the synthetic bones to be 13 mm, the normalized flexural rigidity agrees well with the results from human specimens. Furthermore, our test protocols were only uniaxial and quasi-static. This is a major difference to real world scenarios where forces and moments act about three axes, and particularly during falling over a very short time period [10,11]. Additionally the typical limitations for *in vitro* investigations also apply for our study, as bone remodeling and subsequent processes are not accounted for.

Direct comparison of our findings to previous studies that utilized classical four-point bending tests is limited. The present study used a modified four-point bending setup to apply pure moment bending for the following reasons: (1) this setup prevents torsion, that would otherwise result from non-coplanar support of the specimen, and axial force; (2) highly reproducible specimen positioning is facilitated; and (3) the risk of indenting the human specimens at the bearing, particularly in a high load test, is eliminated.

The results of the axial pull-out test refer to the stability of the cement-implant interface. Therefore we did not test the human specimens with press-fit anchored stem, as this would be limited to early primary stability.

In a finite element study, it was pointed out that the factor of safety against mechanical bone failure of the lower extremity is relatively small, even in load cases for level walking [13]. Compared to previous findings regarding loads at the osseointegrated prosthesis fixation of the lower extremity during falling [12], the present results illustrate the high risk of fracture. Welke et al. reported bending moments at the osseointegrated prosthesis fixation of up to 176 Nm for forward falling on one knee and 187 Nm for falling backwards. Therefore our findings underline the need for the use of a safety element to prevent such fractures as they are related to complicated revision and in the long term a substantial loss of quality of life. The ultimate bending moments observed are considerably below simulated resulting peak moments for dynamic falling scenarios [12], and it should be considered that the duration of load application during falling is range of hundreds of milliseconds.

## Conclusion

The results of this study are of interest with respect to previous findings regarding the load at the interfaces of osseointegrated prosthesis fixation devices, and are crucial for the development of safety devices protecting the bone-implant interface from loading levels that could lead to failure. Future studies may focus on more realistic *in vitro* experiments of falling scenarios to put simulation results into context. Additionally one might investigate the effect of different transfemoral and transhumeral amputation heights on flexural rigidity, fracture modes and ultimate bending moment.

## Abbreviations

OPRA: Osseointegrated Prosthesis for the Rehabilitation of Amputees; DEXA: Dual-Energy X-ray Absorptiometry; CV: coefficient of variance.

## Competing interests

The authors declare that they have no competing interests.

## Authors’ contributions

The following authors have designed the study (BW, CH, TC), performed the experiments, gathered and analyzed the data (BW, MF, MS, TC), written the initial draft (BW, CH, MS, TC), and ensured the accuracy of the data and analysis (BW, CH, MF, MS, TC). All authors read and approved the final manuscript.

## References

[B1] Hagberg K Brånemark R One hundred patients treated with osseointegrated transfemoral amputation prostheses–rehabilitation perspective J Rehabil Res Dev 2009 46 1 16 19675986

[B2] Sullivan J Uden M Robinson KP Sooriakumaran S Rehabilitation of the trans-femoral amputee with an osseointegrated prosthesis: the United Kingdom experience Prosthet Orthot Int 2003 27 114 120 14571941 10.1080/03093640308726667

[B3] Haraldson T Carlsson GE Bite force and oral function in patients with osseointegrated oral implants Scand J Dent Res 1977 85 200 208 265612 10.1111/j.1600-0722.1977.tb00554.x

[B4] Branemark P-II Osseointegration and its experimental background J Prosthet Dent 1983 50 399 410 6352924 10.1016/s0022-3913(83)80101-2

[B5] Aschoff HH Clausen a Tsoumpris K Hoffmeister T [Implantation of the endo-exo femur prosthesis to improve the mobility of amputees] Oper Orthop Traumatol 2011 23 462 472 22083046 10.1007/s00064-011-0054-6

[B6] Aschoff HH Kennon RE Keggi JM Rubin LE Transcutaneous, distal femoral, intramedullary attachment for above-the-knee prostheses: an endo-exo device J Bone Joint Surg Br 2010 92 Suppl 2 180 186 American volume 10.2106/JBJS.J.00806 21123601

[B7] Hagberg K Branemark R Gunterberg B Rydevik B Osseointegrated trans-femoral amputation prostheses: prospective results of general and condition-specific quality of life in 18 patients at 2-year follow-up Prosthet Orthot Int 2008 32 29 41 18330803 10.1080/03093640701553922

[B8] Tillander J Hagberg K Hagberg L Brånemark R Osseointegrated titanium implants for limb prostheses attachments: infectious complications Clin Orthop Relat Res 2010 468 2781 2788 20473597 10.1007/s11999-010-1370-0 PMC2939339

[B9] Blumentritt S Schmalz T Jarasch R The Safety of C-Leg: Biomechanical Tests JPO Journal of Prosthetics and Orthotics 2009 21 2 15

[B10] Frossard LA Load on osseointegrated fixation of a transfemoral amputee during a fall: Determination of the time and duration of descent Prosthet Orthot Int 2010 34 472 487 20961183 10.3109/03093646.2010.520057

[B11] Frossard LA Tranberg R Haggstrom E Pearcy M Branemark R Load on osseointegrated fixation of a transfemoral amputee during a fall: loading, descent, impact and recovery analysis Prosthet Orthot Int 2010 34 85 97 20196690 10.3109/03093640903585024

[B12] Welke B Schwarze M Hurschler C Calliess T Seehaus F Multi-body simulation of various falling scenarios for determining resulting loads at the prosthesis interface of transfemoral amputees with osseointegrated fixation Journal of orthopaedic research: official publication of the Orthopaedic Research Society 2013 31 1123 1129 23494733 10.1002/jor.22329

[B13] Tomaszewski PK Verdonschot N Bulstra SK Verkerke GJ A comparative finite-element analysis of bone failure and load transfer of osseointegrated prostheses fixations Ann Biomed Eng 2010 38 2418 2427 20309731 10.1007/s10439-010-9966-9 PMC2882037

[B14] Lunow C Staubach K-H Aschoff H-H [Endo-exo femoral prosthesis: clinical course after primary implantation of an intramedullary percutaneous endo-exo femoral prosthesis following upper leg amputation] Der Unfallchirurg 2010 113 589 593 20544174 10.1007/s00113-009-1737-4

[B15] Bunke S Wulff W Kraft M Analysis of risks in using a bone-anchored limb prosthesis Orthopädie-Technik 2010 11 800 804

[B16] Chiu J Robinovitch SN Prediction of upper extremity impact forces during falls on the outstretched hand J Biomech 1998 31 1169 1176 9882050 10.1016/s0021-9290(98)00137-7

[B17] DeGoede KM Shton-Miller JA Fall arrest strategy affects peak hand impact force in a forward fall J Biomech 2002 35 843 848 12021005 10.1016/s0021-9290(02)00011-8

[B18] Grundei H Von Stein T Schulte-Bockhof D Kausch C Gollwitzer H Gradinger R Die Endo-Exo-Femurprothese - Update eines Versorgungskonzeptes zur Rehabilitation von Oberschenkelamputierten Orthopädie-Technik 2009 3/09 143 149

[B19] Frossard L Stevenson N Smeathers J Häggström E Hagberg K Sullivan J Ewins D Gow DL Gray S Brånemark R Monitoring of the load regime applied on the osseointegrated fixation of a trans-femoral amputee: a tool for evidence-based practice Prosthet Orthot Int 2008 32 68 78 18330805 10.1080/03093640701676319

[B20] Gardner MP Chong ACM Pollock AG Wooley PH Mechanical evaluation of large-size fourth-generation composite femur and tibia models Ann Biomed Eng 2010 38 613 620 20049637 10.1007/s10439-009-9887-7

[B21] Soininvaara T a Harju K a L Miettinen HJ a Kröger HPJ Periprosthetic bone mineral density changes after unicondylar knee arthroplasty Knee 2013 20 120 127 23154036 10.1016/j.knee.2012.10.004

[B22] Sievänen H Kannus P Oja P Vuori I Precision of dual energy x-ray absorptiometry in the upper extremities Bone Miner 1993 20 235 243 8490327 10.1016/s0169-6009(08)80004-9

[B23] Heiner AD Structural properties of fourth-generation composite femurs and tibias J Biomech 2008 41 3282 3284 18829031 10.1016/j.jbiomech.2008.08.013

